# Unified influence estimation through multi-hop reinforcement and diversity-weighted fusion in weighted complex networks

**DOI:** 10.1038/s41598-026-52304-1

**Published:** 2026-05-18

**Authors:** Ramya D. Shetty, Rashmi M, Keerthan Kumar T G, Shrutilipi Bhattacharjee, K. Jayashree Hegde, Vasudeva, Rashmi Naveen Raj

**Affiliations:** 1 Manipal Institute of Technology, Manipal Academy of Higher Education, Manipal, India; 2 Department of Information Science and Engineering, Siddaganga Institute of Technology, Tumkur, Karnataka India; 3 Department of Information Technology, National Institute of Technology Karnataka, Surathkal, Mangalore, Karnataka India; 4 Department of Information Science and Engineering, Nitte (Deemed to be University), NMAM Institute of Technology (NMAMIT), Nitte, Karnataka India

**Keywords:** Complex network, Disease transmission, Edge weights, Weighted degree, Weighted networks, Industry innovation and infrastructure (SDG 9), Sustainable cities and communities (SDG 11), Engineering, Mathematics and computing, Physics

## Abstract

By depicting items as nodes and their connections as links, networks or graphs attract more attention in complex systems as a way to simulate real-world interactions. Finding influential nodes in human contact networks or other social networks is essential to comprehending disease transmission, which is dependent on the frequency and intensity of contact. Nevertheless, the majority of current research ignores the variability of real-world interactions in favour of uniform connection strength. To address this gap, we propose a unified influence estimation model (uiem) that integrates multi-hop diffusion, local structural reinforcement, and interaction diversity into a single adaptive framework. The model constructs a weighted graph where edge weights reflect interaction frequency/ real weights based on the different scenarios. One-hop and two-hop components capture direct and indirect diffusion influence, while the local structural reinforcement index (lsri) quantifies a node’s connectivity strength and connections within its neighborhood. Additionally, a diversity-weighted fusion (dwf) mechanism combines weighted degree and local clustering entropy (lce) to balance structural intensity and interaction diversity. Experimental results on multiple human contact networks and social networks demonstrate that uiem outperforms existing methods, effectively identifying structurally and functionally influential nodes and providing deeper insights into the dynamics of real-world contact-based spreading processes.

## Introduction

A complex network is an abstract representation of a complex system in the real-world where the elements and interactions among them are represented as nodes and edges, respectively. A vast array of real-world systems, such as those related to biology, society, technology, and infrastructure, can be conceptualized as large complex networks. In these networks, objects are vertices and relationships between objects serve as edges^1–3^. The statistical characteristics of the network nodes and interconnections frequently have a substantial impact on the behavior of the complex network. The complex network is characterized by a non-homogeneous topological structure, which requires each node to maintain a unique status^4^. The network is significantly influenced by a limited number of nodes, which are essential to the network. In contrast, most nodes have minimal or no impact on the network. Consequently, identifying critical nodes through quantitative methods and utilizing their properties are of theoretical significance and promising application^5^.

Identifying influential nodes in complex networks is essential for a variety of real-world applications^1,6–8^. For instance, the prevention of the dissemination of rumors and infections^9–11^, the targeting and discovery of drugs in biomedicine^12^, the facilitation of the rapid dissemination of practical information within the network^13^, evidence theory^14^, and drug networks^15^, etc. These examples highlight the broad applicability of influential node detection in complex network analysis. However, in this work we specifically focus on human contact networks, where edge weights represent the frequency or strength of interactions between individuals. The behavior of critical/influential nodes, which functions as the source of the propagation^16^, has a more rapid diffusion effect on the entire network during the spreading process, in contrast to that of normal nodes^17^. Finding influential elements in complex networks can reveal substantial patterns within the system. Recognizing these features not only enhances our understanding of network functionality but also facilitates the identification of optimal solutions for network issues. As a result, it is important to improve detection methods and evaluate key spreaders in order to get accurate results.

A weighted network is a type of social network analysis graph that has weights on the edges. These weights represent the relationship strength between the nodes. Compared to unweighted networks, which consider all connections equal, weighted networks offer a more precise representation of social structures^18^. Weighted networks are often used to study social networks, as they provide a more detailed picture of social interactions than unweighted networks. Weighted networks are used to mimic the structure of real social networks in to model social dynamics, such as how people develop ideas and share information.

Influential node identification in weighted networks is essential for applications such as influence maximization and rumor control, etc. By assigning weights to social links based on the intensity of pairwise relations, it is possible to more accurately identify significant actors in social networks^19^. Literature attempts to rank nodes based on their propagation capabilities through weighted-degree and weighted K-shell methods. Due to various real-world applications of weighted networks, research on this is a very hot trend among researchers. This paper proposes a method for identifying influential nodes in weighted networks by assessing the network’s edge strength and node degree. The key contributions of the proposed work are as follows:

- A comprehensive *unified influence estimation model (uiem)* is proposed that integrates multi-hop diffusion dynamics, local structural reinforcement, and interaction diversity to estimate node influence in weighted complex networks.
- Human contact networks are modeled as *weighted graphs*, where edge weights derived from interaction frequencies capture the heterogeneity of real-world contact intensity.
- The *local structural reinforcement index (lsri)* is introduced to quantify a node’s embeddedness by combining degree-based and weight-based neighborhood connectivity.
- A novel *diversity-weighted fusion (dwf)* mechanism is formulated to adaptively balance connectivity strength and interaction diversity through an entropy-driven weighting scheme.
- Experimental evaluation across multiple human contact networks/ various complex weighted networks demonstrates that the proposed uiem consistently outperforms state-of-the-art centrality methods.

Additionally, the proposed work would contribute to SDG 9 by offering a cutting-edge, innovative network analytics framework, and SDG 11 by enhancing community-level preparedness and response to epidemic.

The rest of the sections are organized in the following manner. Related research on identifying influential nodes is discussed in “Related works” section. The motivation and research gaps stated in “Motivation and research gaps” section. Further, proposed methodology for identifying important nodes considering weights and other structural network properties are introduced in “Proposed methodology” “ section. The experimental configuration, including the dataset statistics are detailed in “Experimental setup”. The experimental results are presented in “Results and discussions” section. Finally, “Conclusions” section concludes the study and outlines potential directions for future research.

## Related works

In recent years, numerous methods have been proposed for identifying influential nodes in complex networks, addressing both weighted^20^ and unweighted networks^21^. The literature groups these existing approaches in different ways, namely structure-based, Eigenvector-based, local information-based, global-information-based, and semi-local information-based approaches^7,22^. This section provides a concise overview of some algorithms for identifying influential nodes.

Network topology significantly influences node importance, and many methods for identifying influential nodes rely primarily on structural information. Classical centrality measures such as Degree Centrality (DC), Closeness Centrality (CC)^23^, Betweenness Centrality (BC)^24^, and K-shell decomposition^25^ have been widely used to detect influential nodes in networks. Degree Centrality quantifies node importance based on the number of direct connections a node has with other nodes^23^. Betweenness Centrality measures the extent to which a node lies on the shortest paths between other nodes, reflecting its role in controlling information flow within the network^24^. Closeness Centrality evaluates how close a node is to all other nodes in the network by considering the shortest path distances^23^. K-shell centrality identifies influential nodes by iteratively removing low-degree nodes and assigning nodes to shells based on their connectivity^25^. Although these measures provide valuable insights into node influence, they also have certain limitations. Degree Centrality relies only on local information and may overlook globally influential nodes^26^. Betweenness and Closeness centralities capture global structural information but are computationally expensive, especially for large-scale networks^26^. In addition, Closeness Centrality may fail to identify locally influential nodes within communities^27^. K-shell decomposition can assign identical shell values to multiple nodes, making it difficult to distinguish their relative importance^28^.

While the above measures are primarily designed for unweighted networks, many real-world networks contain weighted edges that represent the strength or frequency of interactions. To address this limitation, weighted variants of classical centrality measures have been proposed. Weighted Degree Centrality (wdc)^29^ extends degree centrality by incorporating edge weights to reflect the intensity of node interactions. Similarly, weighted Betweenness Centrality (wbc)^29^ considers edge weights while computing shortest paths, allowing nodes that control high-weight communication paths to be identified. Weighted Closeness Centrality (wcc)^29^ measures the proximity of nodes by accounting for weighted shortest path distances.

Although these weighted measures capture interaction strength more effectively than their unweighted counterparts, they still have certain limitations. For instance, weighted degree primarily focuses on direct connections and may ignore broader structural influence, while weighted betweenness and closeness centralities remain computationally expensive for large-scale networks. These limitations motivate the development of more effective influence estimation methods for weighted complex networks.

Eigenvector-based centrality^30^ metrics are a powerful tool in network analysis, providing a deeper understanding of the significance of nodes based on their connections. This metric allocates comparative scores to each node in the network, giving greater weight to connections with high-scoring nodes compared to those with low-scoring ones. Several variants of Eigenvector centrality are found in literature, including Improved Eigenvector-based Centrality Measures (IECM)^31^, Page Ranking, etc. IECM identifies influential nodes in temporal networks by considering the coupling strength between proximity layers. Google initially developed PageRank as a centrality measure to rank web pages according to their significance. It works according to the premise that a page is deemed significant if it is linked to other significant pages^32^. Although Eigenvector-based centrality measures are effective in finding influential nodes, they include a high level of computational complexity as the network size increases, localization issues, sensitivity to network structure, and limited applicability in more complex network types^33^.

Local centrality metrics are essential for finding significant nodes in complex networks because they are computationally efficient and excellent at capturing local network features. Some local centrality measures, such as degree centrality (DC), overlap with structural measures. The local centrality measure of a node focuses on its local topology. In addition to DC, several classic local centrality measures exist in the literature, including H-index^34^, clustering coefficient^35^, etc. A node’s H-index is the maximum value obtained from all its neighbors with degrees having a value of *h* or more. H-Index is simple to calculate but often assigns the same value to many nodes, limiting its ability to distinguish between nodes with similar influence levels^36^. The clustering coefficient of a vertex is equivalent to the ratio of the number of actual connections among its neighbors to the number of conceivable connections. Though the clustering coefficient captures the local connectivity and is adaptable to weighted networks, it demands high computation and is sensitive to network changes^37^.

The global centrality measures offer a comprehensive understanding of the significance of nodes by considering the network’s overall structure and connectivity patterns. Some of these measures overlap with structural measures as these consider the overall structure of the network, for instance, Betweenness centrality (BC), Closeness Centrality (CC), and so on. In addition, Eigenvector centrality is also considered a global centrality measure. In general, these measures are more precise in ranking influential nodes than local ones, as they consider the entire network’s topology^38,39^. However, the computation of global centrality^40,41^ measures necessitates collecting comprehensive network information, which is computationally costly and impractical for large-scale networks. In addition, the global measures are not appropriate for dynamic networks with a topology that changes over time, as they impose significant computational demands^39^.

Semilocal information-based centrality measures^42–44^ are intended to assess the significance of nodes in a network by considering their local and limited global information. These measures are designed to balance the comprehensive insight offered by global centrality measures and the computational efficiency and simplicity of local centrality measures^43^. Although semi-local measures are more efficient than global ones, they are still more complex than solely local ones, which may be a disadvantage in large networks. Most of the methods discussed so far are designed to identify influential nodes by assigning equal strength to all edges in the network. Since edge strength is crucial in real-world networks, the fundamental techniques for unweighted networks are expanded to weighted networks to identify critical nodes. Several works are discussed below determine influential nodes by evaluating the intensity of their edges.

The weighted degree centrality (wdc) measure is calculated by adding the weights of the edges connected to a node^45^. It represents the total number of connections that a node has. Although it is straightforward to calculate and interpret, it may fail to account for nodes essential for connectivity but with lesser weights. In contrast, weighted centrality of the relation (wbc) is an improved version of the conventional centrality of the relationship^46^. The wbc measure is designed to determine the number of shortest paths that pass through a node, the edge weights being considered. The traditional CC is extended to weighted networks in the form of a weighted closeness centrality (wcc)^47^. This measure evaluates the speed with which a node can access all other network nodes, considering the weights of the edges.

The weighted eigenvector centrality (wec)^48^ measure assesses the influence of a node by weighing the influence of its neighbors according to the intensity of their connections. It evaluates both the quantity and quality of connections. The weighted K-shell centrality (wks)^49,50^ computes the centrality considering the weights of the edges. This method improves the overall effectiveness of network analysis tasks and improves the identification of influential spreaders. However, it introduces more computational complexity and places many nodes in the same shell. It is evident from the literature review that the majority of the extant methods are constrained by their high computation requirements and their use of unweighted networks. To identify the most influential nodes in weighted networks, we suggest a novel technique that considers the weights of edges and the strength of nodes.

Further, studies have highlighted the importance of contact-network-based modeling for understanding disease transmission dynamics. For instance, Wu et al.^51^ constructed a temporal contact graph using large-scale smartphone-based contact tracing data to analyze the evolution of COVID-19 spread. Their study demonstrated that temporal contact patterns can reveal important epidemic indicators and provide valuable insights into population-level transmission dynamics. Similarly, Kumar and Panda^52^ proposed an improved WVoteRank method to identify influential spreaders in weighted complex networks by incorporating extended neighborhood information. Their approach emphasized the importance of identifying structurally significant nodes that can accelerate information or disease propagation in complex networks. These studies demonstrate that both contact network structure and influential node identification play a critical role in understanding and controlling epidemic spreading processes. Motivated by these findings, the present work focuses on modeling weighted human contact networks and proposes the uiem framework to more accurately estimate influential nodes responsible for disease propagation.

## Motivation and research gaps

Despite significant advances in network science, accurately identifying potential nodes in weighted complex networks remains to be an ongoing challenge. Traditional centrality measures, though conceptually simple and computationally efficient, often overlook the heterogeneous intensity of real-world interactions (Example-1: In a social network, some people interact daily (strong connection), while others rarely interact (weak connection). Example-2: In a transportation network, some routes have high traffic (strong link), while others are rarely used (weak link)). In many real systems such as human contact networks, transportation systems, and information diffusion networks, the strength of connections (edge weights) plays a crucial role in determining influence propagation. Nevertheless, a number of current models still assume uniform link strength, especially in unweighted or undirected representations, which restricts their capacity to accurately depict diffusion dynamics.

Additionally, the existing literature highlights the following study gaps and limitations:

- *Neglect of edge-weight heterogeneity* The majority of traditional and even some modern centrality-based techniques approach networks as unweighted, neglecting to take into account the fluctuating frequency or intensity of node interactions.
- *Limited integration of structural and weighted properties* Incomplete influence estimate results from approaches that take edge weights into account because they frequently ignore the interaction between structural connectivity (degree-based) and weighted intensity (strength-based) variables.
- *Insufficient modeling of multi-hop influence* Many algorithms capture only direct (one-hop) influence, while indirect (multi-hop) effects essential for understanding real diffusion processes remain underrepresented.
- *Lack of adaptive fusion strategies* Existing weighted methods typically apply fixed combinations of network attributes, without dynamically balancing structural intensity and interaction diversity.
- *Computational complexity in large networks* Several global or eigenvector-based centrality measures require complete network information and exhibit scalability issues when applied to large, real-world graphs.

To overcome these limitations, the proposed *unified influence estimation model (uiem)* introduces a holistic framework that jointly captures weighted connectivity, multi-hop propagation, local structural reinforcement, and interaction diversity. Specifically, UIEM incorporates edge-weight heterogeneity through weighted degree and weight-normalized influence propagation (addressing Gaps 1 and 2), models extended diffusion pathways via a two-hop influence mechanism (Gap 3), employs an adaptive fusion strategy that balances structural intensity with entropy-driven diversity (Gap 4), and relies entirely on local and semi-local computations to maintain scalability on large networks (Gap 5). This integrated approach enables a more realistic and computationally efficient estimation of node influence in weighted complex networks.

## Proposed methodology

This section presents the step-by-step procedure of the proposed unified influence estimation model, which integrates multi-hop connectivity, degree-based neighborhood weighting, and entropy-driven diversity measures to estimate node influence in weighted complex networks. The overall framework consists of several sequential stages, from graph construction to final ranking.

### Graph construction

The interaction dataset is represented as an undirected weighted graph $G = (V, E)$, where each node $v \in V$ denotes an individual and each edge $e = (i, j) \in E$ represents an interaction between nodes $i$ and $j$ in contact networks. The weight $w_{ij}$ of an edge corresponds to the frequency of interactions between the node pair, derived from the raw interaction data. When multiple interactions occur between the same pair, their frequencies are accumulated to form the final edge weight. We consider two types of network constructions in this work: (i) Weighted networks derived from interaction frequency, where edge weights are computed directly using Algorithm 1; and (ii) Pre-existing weighted networks, where the raw data already provide edge weights, eliminating the need for explicit weight derivation.

Intuition: Real-world interactions do not all carry the same importance. Some people interact frequently, others rarely; some connections are strong, some weak. By constructing a weighted network, we ensure that strong, frequent, or meaningful interactions contribute more weak or occasional interactions contribute less. This step allows the influence model to operate on a realistic representation of the strength of the interaction, rather than treating each connection equally. Present study focuses on undirected weighted networks, as the datasets considered represent human contact interactions that are typically treated as mutual.

**Algorithm 1:**
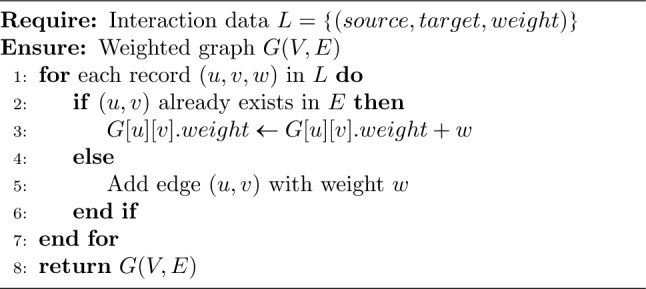
Graph construction and edge weight aggregation

### Node-level structural properties

Two baseline structural properties are computed for each node $i \in V$:

- *Degree* $d_i$: Number of edges incident on node $i$.
- *Weighted degree* $wd_i$: Sum of edge weights connected to node $i$, defined as
  1 $$\begin{aligned} wd_i = \sum _{j \in N(i)} w_{ij} \end{aligned}$$

Intuition: Before computing influence, we first need to understand a node’s basic structural role in the network, such as: how many neighbors it has (degree), how strongly it is connected (weighted degree). These structural features serve as the foundation on which all later influence calculations are built. They help answer: How well-connected is a node?, How strong are its ties?.

### One-hop influence ($I_1$)

The one-hop influence captures the direct impact of a node on its immediate neighbors, considering edge weights and the connectivity of those neighbors. It is defined as:

2 $$\begin{aligned} I_1(i) = \sum _{j \in N(i)} \frac{w_{ij}}{1 + d_j} \end{aligned}$$

This ensures that influence from a neighbor diminishes when the neighbor is highly connected.

Intuition: Not all neighbors contribute equally to a node’s influence. $I_{1}$ captures the immediate influence potential of a node by considering: the strength of its direct connections and how influential its neighbors already are. This reflects the common idea: “A person is influenced most strongly by the people directly connected to them”. $I_{1}$ quantifies local spreadability.

### Two-hop influence ($I_2$)

To account for indirect influence propagation through two-hop connections, the two-hop influence component aggregates the weighted paths of length two:

3 $$\begin{aligned} I_2(i) = \sum _{j \in N(i)} \sum _{\begin{array}{c} k \in N(j) \\ k \ne i \end{array}} \frac{w_{ij} \cdot w_{jk}}{w_{\max } (1 + d_j)(1 + d_k)} \end{aligned}$$

where $w_{\max }$ is the maximum edge weight in the network. The normalization by $w_{max}$ is introduced to control the magnitude of the weighted path contribution and to ensure numerical stability in the computation of two-hop influence scores. Since the product $w_{ij} \cdot w_{jk}$ may become large in networks with high interaction frequencies, dividing by $w_{max}$ scales the two-hop contribution to a bounded range and prevents dominance of extremely high-weight edges in the influence calculation.

Intuition: Influence rarely stops at immediate neighbors. Many diffusion processes, such as rumor spread, disease transmission, and information flow, reach friends of friends. $I_{2}$ models this second layer, capturing indirect but meaningful influence. It helps identify nodes that may not have strong immediate connections but have a high influence through their extended network.

### Local structural reinforcement index (lsri)

(a) Topological Reinforcement Component: To represent unweighted structural cohesion, the following measure is used:

4 $$\begin{aligned} lsri_{deg}(i) = \sum _{j \in N(i)} \frac{ \delta _i + \delta _j }{ \sigma _{\delta } } \end{aligned}$$

where:

- $N(i)$ denotes the set of neighbors of node $i$,
- $\delta _i$ and $\delta _j$ represent the degree of nodes $i$ and $j$, respectively,
- $\sigma _{\delta }$ is the standard deviation of node degrees across the network.

This component captures the extent to which a node $i$ and its neighbors collectively contribute to the local connectivity backbone.

(b) Weighted Reinforcement Component: To incorporate the cumulative intensity of connections, a weighted variant is defined as:

5 $$\begin{aligned} lsri_{wgt}(i) = \sum _{j \in N(i)} \frac{ wd_i + wd_j }{ \sigma _{w} } \end{aligned}$$

where, $wd_i$, $wd_j$, and $\sigma _{w}$ denotes the weighted degree of node $i$, $j$, and the standard deviation of weighted degrees in the network, respectively.

Together, these two components describe how locally cohesive and interaction-intensive a node’s neighborhood is. Nodes with high $lsri_{deg}$ and $lsri_{wgt}$ values serve as *local reinforcement hubs*, bridging structurally dense and strongly connected regions of the graph.

Intuition: Influence is not only about how many neighbors you have, but also how those neighbors support or reinforce each other. The lsri measures the cohesiveness around a node: If a node is in the middle of a tight-knit group, it gets a lot of reinforcement. Low reinforcement happens when the neighbors are loosely connected or spread out. This step answers the following question: “Is the node supported by a strong local structure, or is it standing alone?”.

### Local clustering entropy (lce)

To quantify the diversity of interaction strength around a node, we employ an entropy-based measure:

6 $$\begin{aligned} lce(i) = - \sum _{j \in N(i)} p_{ij} \log (p_{ij} + \epsilon ) \end{aligned}$$

where $p_{ij} = \frac{w_{ij}}{\sum _{k \in N(i)} w_{ik}}$ and $\epsilon = 10^{-12}$ is a small numerical constant introduced to ensure computational stability and avoid floating-point precision issues in logarithmic calculations. Higher entropy indicates more uniformly distributed interactions across neighbors, reflecting diverse connectivity.

Intuition: Not all influential nodes belong to dense clusters. Some important nodes connect to diverse and heterogeneous neighborhoods. The lce measures diversity of interactions, not just density. High entropy connections are spread across different types of neighbors. Low entropy connections are concentrated within a homogeneous group. This helps identify nodes that: bridge communities, connect diverse groups, facilitate cross-community information flow. These nodes are often hidden influencers.

### Diversity-weighted fusion component (dwf)

The proposed method employs a DWF component to integrate connection strength and interaction diversity into a single adaptive metric. This approach makes sure that nodes that are very connected and have different ways of interacting receive equivalent levels of influence reinforcement.

7 $$\begin{aligned} dwf(i) = \alpha \cdot wd_{i} + (1 - \alpha ) \cdot \mathcal {H}(i) \end{aligned}$$

where:

- $wd_{i}$ represents the weighted degree (cumulative interaction strength) of node $i$,
- $\mathcal {H}(i)$ denotes the local clustering entropy (lce), which quantifies the diversity of edge-weight distribution within the neighborhood of node $i$,
- $\alpha \in [0, 1]$ is a balancing coefficient that determines the relative importance of structural intensity versus entropy-driven diversity.

A moderate value of the fusion parameter $\alpha$ is used to balance the contribution of weighted degree and entropy components in the diversity-weighted fusion (dwf). Instead of fixing $\alpha$ to a constant value, this study determines the optimal value through sensitivity analysis by varying $\alpha$ from 0 to 1. The best-performing $\alpha$ value for each dataset is selected and used in the final influence estimation (Please refer to Table 1), ensuring adaptability across different network structures.

**Fig. 1:**
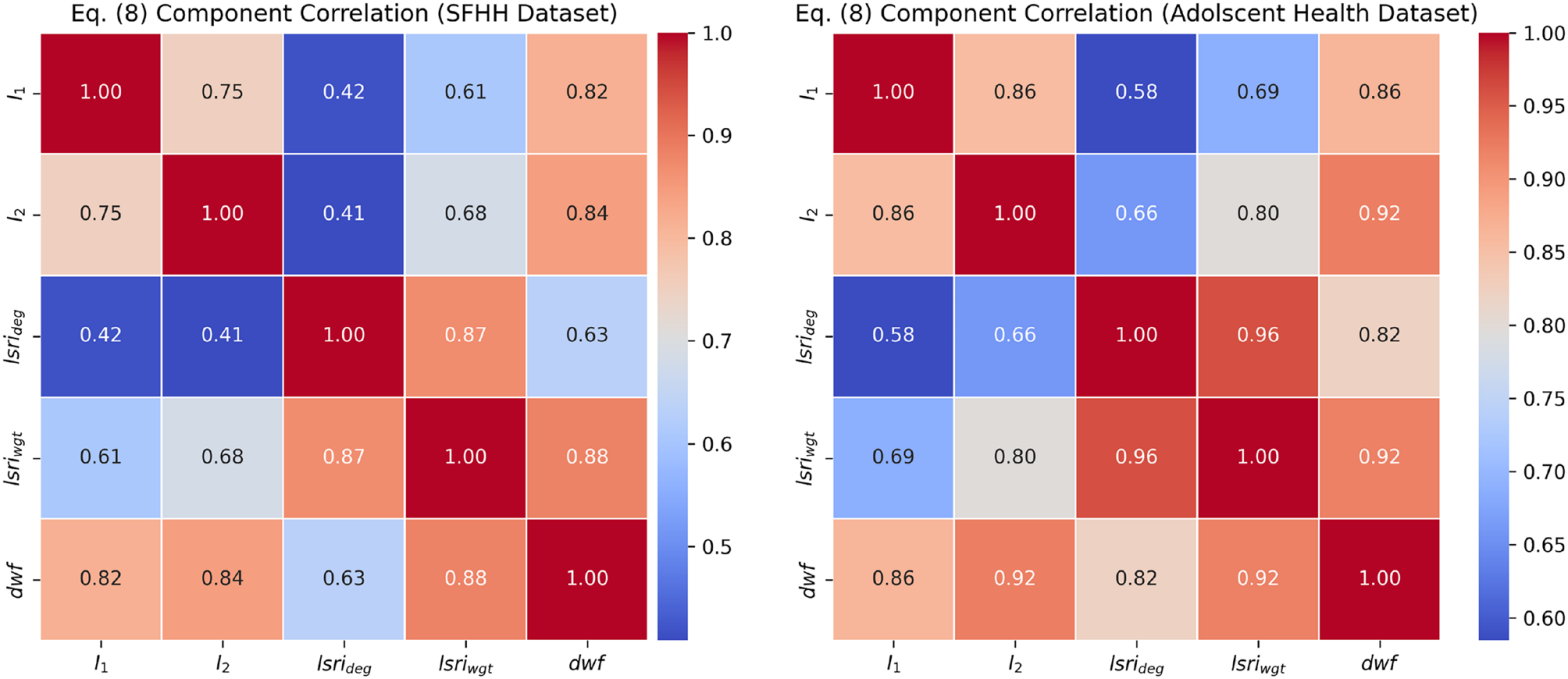
Correlation analysis of the components used in Equation (8) across two datasets.

**Fig. 2:**
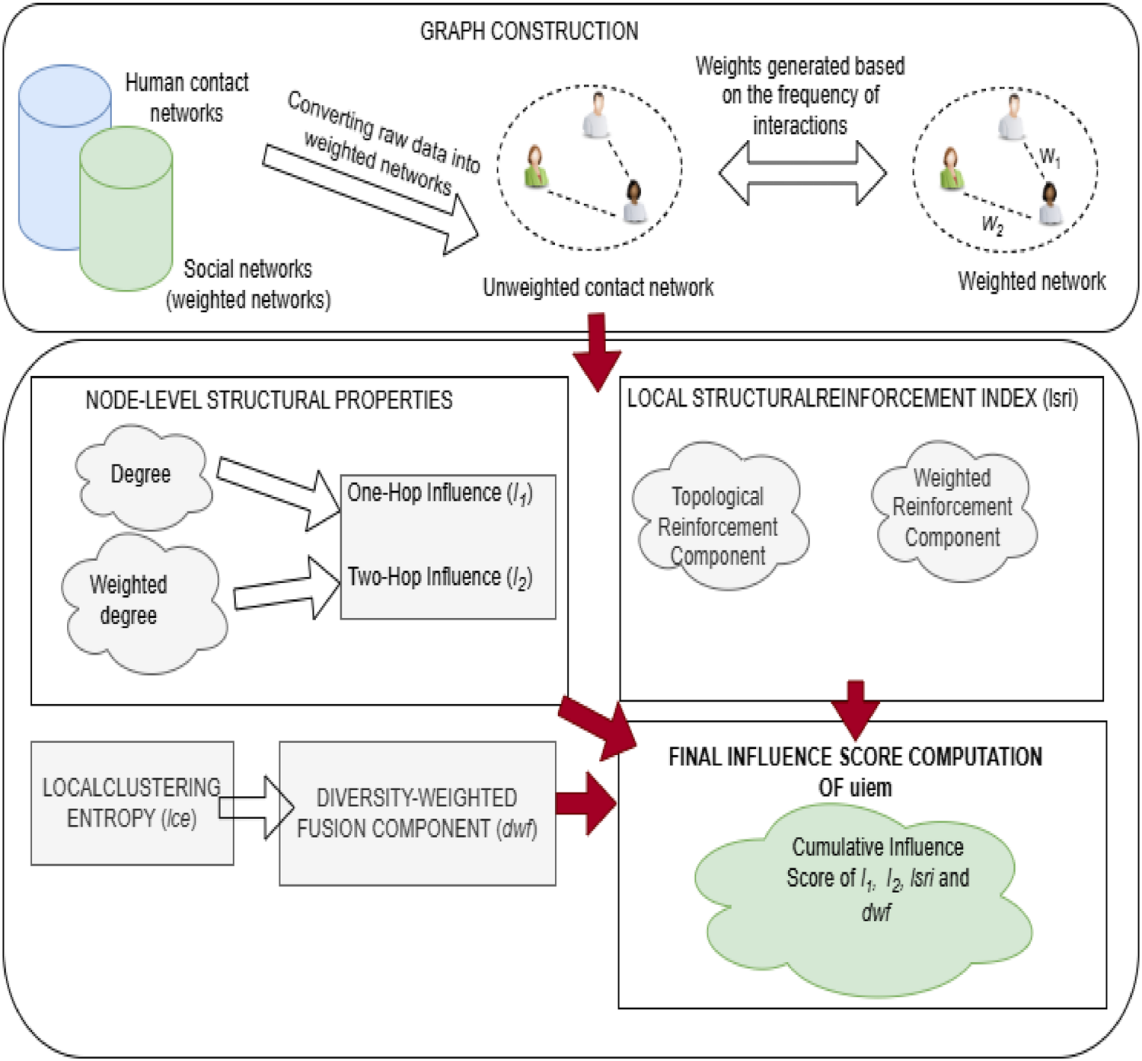
Architecture diagram explaining uiem different stages.

### Final influence score computation of uiem

The overall influence score of the uiem for each node integrates all the above mentioned structural and diffusion-based components:

8 $$\begin{aligned} uiem(i) = \frac{I_1(i)}{\sigma _{I_1}} + \frac{I_2(i)}{\sigma _{I_2}} + \frac{lsri_{deg}(i)}{\sigma _{lsri_{deg}}} + \frac{lsri_{wgt}(i)}{\sigma _{lsri_{wgt}}} + \frac{dwf(i)}{\sigma _{dwf}} \end{aligned}$$

Here, $\sigma _{I_1}$, $\sigma _{I_2}$, $\sigma _{lsri_{deg}}$, $\sigma _{lsri_{wgt}}$, and $\sigma _{dwf}$ denote the standard deviations of the corresponding components computed across all nodes in the network. This normalization ensures that all components operate on a comparable scale and prevents any single term from disproportionately influencing the final influence score.

*Discussion* The uiem framework integrates five normalized components: $I_1$, $I_2$, $lsri_{deg}$, $lsri_{wgt}$, and *dwf*, each designed to capture a distinct structural or diffusion characteristic of node influence in weighted networks. Specifically, the One-Hop Influence ($I_1$) captures the direct spreading capability of a node through its immediate neighbors while accounting for the connectivity of those neighbors. The Two-Hop Influence ($I_2$) models indirect propagation potential through second-order neighbors, thereby reflecting diffusion pathways that extend beyond the local neighborhood. The Local Structural Reinforcement components ($lsri_{deg}$ and $lsri_{wgt}$) quantify the structural cohesion and reinforcement within a node’s neighborhood by incorporating both degree-based connectivity and weighted interaction intensity. The Diversity-Weighted Fusion (*dwf*) integrates interaction strength and interaction diversity by combining weighted degree and entropy-based measures, enabling the model to identify nodes that act either as strongly connected hubs or as structurally diverse bridges. Thus, rather than relying on a single dominant factor, the uiem model follows a complementary fusion strategy in which each component captures a different dimension of influence: diffusion potential ($I_1$, $I_2$), structural reinforcement ($lsri_{deg}$, $lsri_{wgt}$), and interaction diversity (*dwf*).

Finally, all nodes are ranked in descending order of their influence scores to obtain the final ranking list. The ranked output is exported for further evaluation and correlation with epidemic-based dynamic simulations such as the W-SIR model. All the above steps are stated in Algorithm 2. All these variations are presented in Fig. 2.

To examine potential redundancy among the components used in Equation (8), we performed a correlation analysis on two real-world social network datasets, namely the *SFHH dataset* and the *Adolescent contact dataset*. The correlation matrices of the five components $I_1(i)$, $I_2(i)$, $lsri_{deg}(i)$, $lsri_{wgt}(i)$, and *dwf*(*i*) are illustrated in Fig. 1. The results show moderate to strong correlations among the structural metrics, which is expected since many centrality-based measures depend on shared topological properties of the network.

For the SFHH dataset, the correlations range approximately from 0.41 to 0.88, while for the Adolescent dataset they range from 0.58 to 0.96. The highest correlation is observed between $lsri_{deg}(i)$ and $lsri_{wgt}(i)$, which is expected because both metrics originate from the local structural influence framework but represent different aspects of network connectivity. These observations confirm that the components provide complementary structural information, thereby supporting their integration in the uiem influence score.

### Computational complexity analysis

The computational complexity of the proposed *uiem* model is primarily influenced by the two-hop influence component. The computation of degree, weighted degree, local structural reinforcement indices, and local clustering entropy each require a single pass over all edges, contributing a linear-time cost of *O*(*m*) per component, where *m* is the number of edges. The one-hop influence $I_1$ also operates in *O*(*m*) time. In contrast, the two-hop influence $I_2$ involves iterating over neighbors of neighbors and requires $O\!\left( \sum _{j \in V} \deg (j)^2\right)$ time, which simplifies to $O(m\Delta )$, where $\Delta$ denotes the maximum node degree. This term becomes the dominant factor in the overall complexity. In dense networks, the worst-case cost may approach $O(n^3)$, while in typical sparse real-world networks where $\Delta$ is comparatively small–the practical complexity reduces to $O(n\Delta )$. The final node ranking step adds an additional $O(n \log n)$ time. Therefore, the overall computational complexity of *uiem* is $O(m\Delta )$ for sparse networks, with the two-hop influence computation representing the principal computational bottleneck.

It is important to note that the proposed method is primarily intended for sparse interaction networks such as social contact networks, which typically exhibit low average degree and limited connectivity density. In highly dense networks (e.g., functional brain networks or financial correlation networks), the two-hop computation may become computationally expensive, and further optimization strategies or approximation techniques may be required. In practical real-world sparse datasets used in this study, the computational cost remains manageable due to the limited neighborhood expansion and localized influence computation.

## Experimental setup

Here, we provide a detailed description of the dataset statistics and the evaluation metrics used to assess the ranking correlation between the W-SIR model and various node-ranking approaches.

### Dataset statistics

The proposed method is assessed in eight distinct publicly available datasets. The network structural properties of all the datasets used in this study are reported in Table 1 and available at the following link: https://networkrepository.com/^53^, and https://www.sociopatterns.org/datasets/. The ICCSS17, SFHH, and Village datasets represent human contact networks in which the raw data contain interaction events with associated timestamps. These networks are converted into weighted graphs using Algorithm 1. In contrast, the remaining datasets are already provided as weighted networks in their raw form and therefore require no additional weight computation.

### Comparison algorithms used for the current study

This section presents a brief overview of the eight comparison algorithms used in our study to evaluate the effectiveness of the proposed approach.

- Weighted degree centrality (wdc) as defined in^29^, measures the strength of a node’s direct interactions by accounting for the total weights of all its immediate connections.
- Weighted Betweenness Centrality (wbc)^29^ defined as, a node’s centrality is determined by how often it lies on the shortest paths between pairs of nodes, with the calculation accounting for the influence of edge weights.
- Weighted closeness centrality (wcc)^29^ measures how efficiently a node can reach all others, defined as the inverse of the total weighted shortest-path distances from that node.
- Weighted eigenvector centrality (wec) evaluates a node’s importance by considering both its connectivity and the influence of its neighbors, incorporating edge weights to reflect the strength of interactions^54,55^.
- Weighted *k*-shell method (wks) proposed in^56^, combines a node’s structural connectivity with the strength of its connections (edge weights) to compute a weighted degree for each node.
- Normalized Strength-Degree centrality (nsd)^57^ quantifies a node’s influence by jointly considering its degree and strength after normalizing them to a unified scale. This measure reflects both how many connections a node has and how strong those connections are.
- Weighted VoteRank (wvr)^58^ identifies multiple influential spreaders in weighted networks by allowing each node to allocate votes to its neighbors based on their degree, voting ability, and edge weights.
- Clustering degree algorithm (cda)^59^ integrates a node’s weighted degree and clustering behavior to compute its clustering-based influence score. It then redistributes influence from neighbors based on edge weights, producing a final ranking of node importance.

**Algorithm 2:**
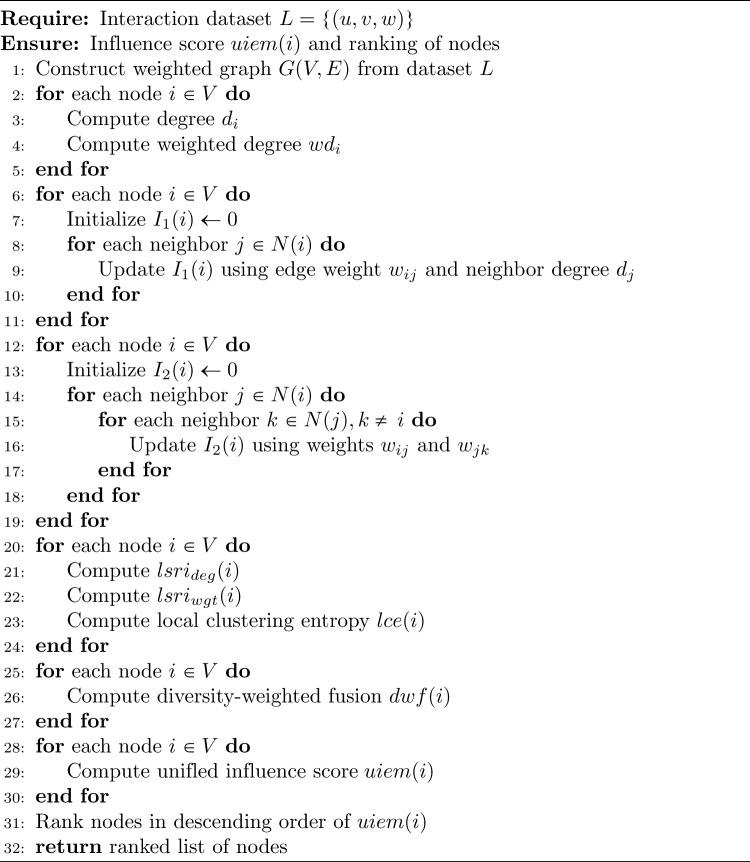
Unified Influence Estimation Model (uiem)

**Table 1:** Key network parameters include the number of vertices (*V*), the number of edges (*E*), the infection probability ($\beta$), threshold value ($\beta _{\text {th}}$) and balancing coefficient ($\alpha$).

Network	*E*	*V*	$\beta _{th}$	$\beta$	$\alpha$
ICCSS17^60^	17327	262	0.007	0.04	0.5
ANU-residence^61^	2672	217	0.048	0.09	0.5
SFHH^62^	9565	403	0.015	0.06	0.9
Caenorhabditis Elegans^61^	2025	453	0.038	0.08	0.5
Japanese Macaques^61^	1187	62	0.026	0.05	0.1
Adolescent Health^61^	12969	2539	0.095	0.10	0.1
High school^61^	366	70	0.107	0.15	0.1
Village^63^	347	86	0.088	0.1	0.5

**Table 2:** The average Kendall’s correlation score analysis between uiem and various comparison algorithms with varying infection probability rate.

Network	$\sigma (\tau _{wdc})$	$\sigma (\tau _{wbc})$	$\sigma (\tau _{wcc})$	$\sigma (\tau _{wec})$	$\sigma (\tau _{wks})$	$\sigma (\tau _{nsd})$	$\sigma (\tau _{wvr})$	$\sigma (\tau _{cda})$	$\sigma (\tau _{uiem})$
ICCSS17	0.8002	0.1099	0.0613	0.7483	0.7421	0.7967	0.8065	0.7837	**0.8463**
ANU-residence	0.7858	0.3908	0.4121	0.8107	0.7918	0.7912	0.5603	0.7956	**0.8633**
SFHH	0.6888	0.1099	0.0613	0.3919	0.6138	0.6798	0.6880	0.6793	**0.7387**
Caenorhabditis Elegans	0.5222	0.5274	0.6049	0.3095	0.6592	0.6116	0.4887	0.3883	**0.7042**
Japanese Macaques	0.6643	0.2155	0.2003	0.6760	0.7502	0.7609	0.6499	0.6747	**0.8336**
Adolescent Health	0.5668	0.3410	0.4421	0.5705	0.6443	0.6536	0.4133	0.5853	**0.7042**
High School	0.7542	0.4434	0.6158	0.7263	0.7728	0.7728	0.4568	0.7022	**0.8429**
Village	0.4972	0.0178	-0.0160	0.1002	0.4562	0.4291	0.4272	0.4317	**0.5935**

Best results are highlighted in bold.

**Table 3:** Sensitivity analysis of fusion parameter $\alpha$ showing Kendall $\tau$ correlation scores of UIEM under the W-SIR model.

Dataset	$\alpha =0.1$	$\alpha =0.3$	$\alpha =0.5$	$\alpha =0.7$	$\alpha =0.9$
SFHH	0.6346	0.6773	0.7001	0.7149	**0.7387**
High School	**0.8429**	0.8302	0.8238	0.8208	0.8150

Best results are highlighted in bold.

### Evaluation metrics

### W-SIR model

The Susceptible-Infected-Recovered (SIR) model^64^ has been used frequently in network studies to investigate the spread of epidemics in recent years. The Weighted SIR^56^ denotes modifications or extensions of the classical SIR model that integrate weights or adjustments to more accurately capture the complexities of real-world epidemiological modeling. In the SIR paradigm, each node of the network is either in the S, I, or R states. Here, S-stands for susceptible but uninfected nodes, I-for infected nodes that can spread the disease to susceptible neighbors with a probability of $\beta$, and R-for infected but recovered individuals with a probability of $\gamma$. The recovery probability $\gamma$ is fixed to 1 for all datasets, meaning that each infected node recovers after one time step, which is a commonly used setting in SIR-based influence evaluation studies. Each simulation is repeated for 1000 independent runs to ensure stability of the spreading results.

The infection rate $\beta$ controls the speed of epidemic spreading in the W-SIR model. Larger values of $\beta$ lead to faster diffusion and infection of more nodes, while smaller values result in slower spreading. The optimal value of $\beta$ for a network is typically determined with respect to the epidemic threshold $\beta _{th}$, which can be approximated as $\beta _{th} \sim \langle k \rangle / \langle k^2 \rangle$, where $\langle k \rangle$ and $\langle k^2 \rangle$ denote the average degree and the second-order average degree of the network, respectively^65^. In practice, the infection rate should be chosen slightly higher than the epidemic threshold (i.e., $\beta > \beta _{th}$) to ensure that the spreading process can propagate effectively through the network. Accordingly, in our experiments, the $\beta$ values were selected to be moderately larger than the corresponding $\beta _{th}$ of each dataset.

### Kendall correlation score

Kendall’s tau coefficient^66^ is employed to evaluate the genuine spreading capacity of nodes about their spreading influence. This assesses the extent of correlation between the ranking method and the one produced by the W-SIR model. This evaluates a collection of matched observations from two distinct variables *X* and *Y*. If the ranks of both elements are in line, a pair of observations $(x_i, y_i)$ and $(x_j, y_j)$ is considered concordant, and this implies that either both $x_i$ and $y_i$ are greater than $x_j$ and $y_j$, respectively, or both $x_i$ and $y_i$ are less than $x_j$ and $y_j$, respectively. If $x_i > x_j$ and $y_i < y_j$, or if $x_i < x_j$ and $y_i > y_j$, they are deemed discordant.

## Results and discussions

To demonstrate the effectiveness of the proposed model, we conducted a series of experiments as outlined below.

### Evaluating Kendall correlation coefficient across various comparison algorithms

In this study, we evaluate the effectiveness of the proposed method against eight established ranking techniques used alongside the W-SIR model. Here, we present two sets of results. Table 2 shows the average Kendall $\tau$ correlation scores based on the results of the W-SIR model, calculated over 1000 simulation runs. Among the conventional methods, wdc and wks exhibit relatively stronger performance compared to wbc and wcc. Further, we include comparisons with recent indexing-based approaches such as nsd, wvr, and cda. However, the proposed method, uiem, consistently outperforms all existing techniques across all networks analyzed in this study. The computation of this metric is shown in Eq. (9).

9 $$\begin{aligned} \sigma (\tau ) = \frac{1}{N} \sum _{i=1}^{N} \tau (\beta _{th} + 0.01 \times i), \end{aligned}$$

where $N=5$ represents the number of incremental steps, with $\beta$ increased by 0.01 at each iteration beyond the threshold.

**Fig. 3:**
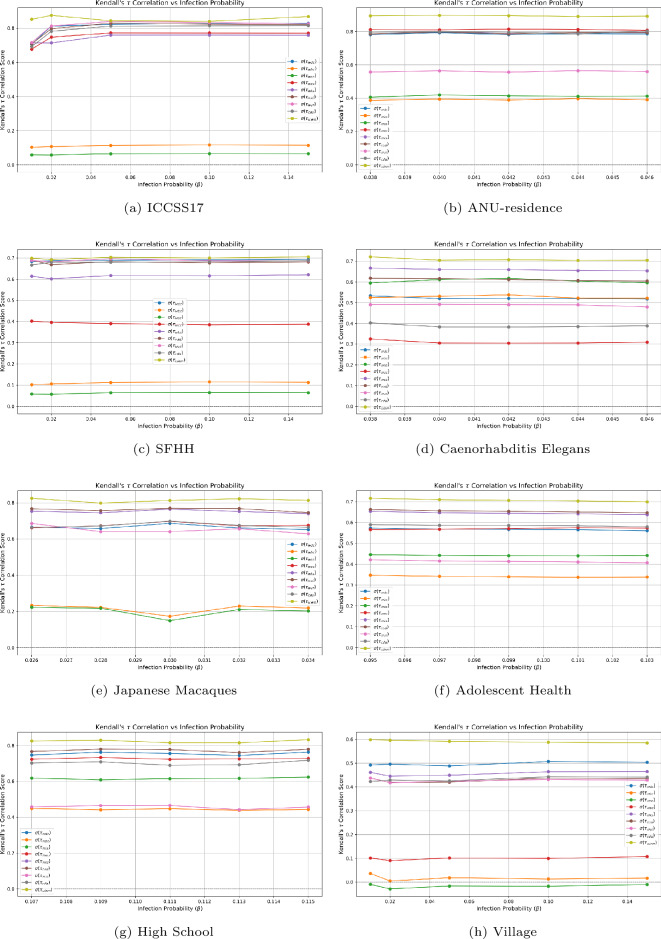
The Kendall $\tau$ correlation scores, plotted on the Y-axis against varying infection probabilities on the X-axis, highlight the performance of various indexing methods alongside the uiem.

In addition, we have presented the results of individual iterations (presented in Figure 3), highlighting the variation in Kendall’s correlation scores corresponding to different infection probability values ($\beta$). In all networks, the proposed uiem method outperforms existing approaches by effectively integrating the various components of the network properties in its evaluation.

To analyze the impact of the diversity-weighted fusion parameter $\alpha$, we conducted a sensitivity analysis by varying $\alpha$ from 0.1 to 0.9. The performance of the proposed uiem model was evaluated using the Kendall $\tau$ correlation coefficient under the W-SIR spreading model across all datasets. Table 2 presents the correlation scores corresponding to the best $\alpha$ value. The best $\alpha$ value for the each of the dataset is mentioned in Table 1

Table 3 shows the variation of Kendall $\tau$ values with different $\alpha$ values. It can be observed that the performance of uiem remains relatively stable across different parameter settings. For some datasets, higher Kendall correlation scores are achieved when $\alpha$ is adjusted, indicating that the contribution of weighted degree and entropy may vary depending on network structure.

**Table 4:** Ranking uniqueness/monotonicity *M*(*I*) measured with respect to various complex networks, where *I* is the Indexing Method Used.

Network	$M(\tau _{wdc})$	$M(\tau _{wbc})$	$M(\tau _{wcc})$	$M(\tau _{wec})$	$M(\tau _{wks})$	$M(\tau _{nsd})$	$M(\tau _{wvr})$	$M(\tau _{cda})$	$M(\tau _{uiem})$
ICCSS17	0.9992	0.9999	0.9600	**1.0**	0.9998	0.9999	0.9962	0.9999	**1.0**
ANU-residence	0.9768	0.9997	0.9969	0.9988	0.9942	0.9945	0.9754	0.9998	**1.0**
SFHH	0.9966	0.9993	0.9929	**1.0**	0.9998	0.9999	0.9982	**1.0**	**1.0**
Caenorhabditis Elegans	0.9903	0.9915	0.9973	0.9990	0.9972	0.9978	0.9765	**0.9990**	**0.9990**
Japanese Macaques	0.9768	**1.0**	0.8662	0.9998	0.9968	0.9976	0.9854	0.9998	**1.0**
Adolescent Health	0.9544	0.9937	0.9997	0.9999	0.9893	0.9926	0.9694	0.9999	**1.0**
High School	0.8804	0.9975	0.9794	**1.0**	0.9622	0.9622	0.9463	0.9974	**1.0**
Village	0.9994	0.8658	0.9967	0.9994	0.9994	0.9994	0.9975	0.9994	**0.9999**

Best results are highlighted in bold.

**Table 5:** Comparative ranking correlation of the proposed uiem approach with existing indexing techniques.

Network	$$\begin{gathered} \tau (wdc, \hfill \\ uiem) \hfill \\ \end{gathered}$$	$$\begin{gathered} \tau (wbc, \hfill \\ uiem) \hfill \\ \end{gathered}$$	$$\begin{gathered} \tau (wcc, \hfill \\ uiem) \hfill \\ \end{gathered}$$	$$\begin{gathered} \tau (wec, \hfill \\ uiem) \hfill \\ \end{gathered}$$	$$\begin{gathered} \tau (wks, \hfill \\ uiem) \hfill \\ \end{gathered}$$	$$\begin{gathered} \tau (nsd, \hfill \\ uiem) \hfill \\ \end{gathered}$$	$$\begin{gathered} \tau (wvr, \hfill \\ uiem) \hfill \\ \end{gathered}$$	$$\begin{gathered} \tau (cda \hfill \\ ,uiem) \hfill \\ \end{gathered}$$
ICCSS17	0.8610	0.1488	0.0990	0.8137	0.7348	**0.9678**	0.8328	0.8416
ANU-residence	0.9126	0.4327	0.3972	0.7231	**0.9291**	0.9287	0.6732	0.8281
SFHH	0.7092	0.5909	0.5741	0.5109	0.8813	**0.9138**	0.7004	0.7441
Caenorhabditis elegans	0.7746	0.4251	0.4022	0.4556	**0.8464**	0.7841	0.5766	0.5986
Japanese Macaques	0.7622	0.1824	0.1611	0.7736	0.8690	**0.8873**	0.7419	0.7747
Adolescent Health	0.7851	0.3590	0.3913	0.5807	0.8902	**0.9005**	0.8352	0.7693
High school	0.8840	0.4864	0.5902	0.6339	0.9193	**0.9193**	0.5610	0.7755
Village	0.6951	− 0.0156	-0.001	0.0662	0.5593	0.7783	0.4241	**0.9053**

Best results are highlighted in bold.

**Fig. 4:**
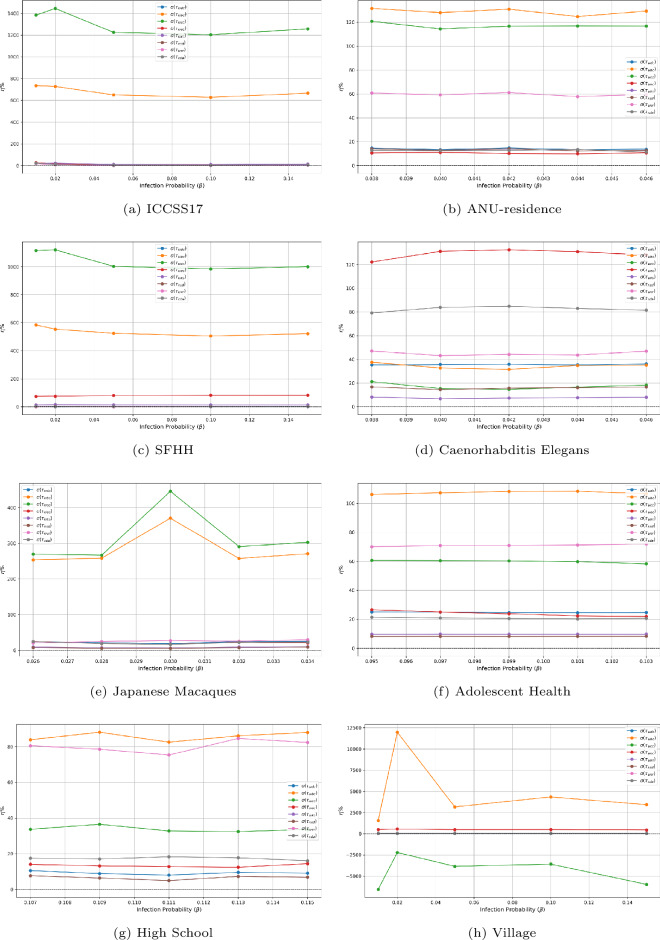
The percentage improvement, denoted as $\eta \%$, represents the performance gain of the proposed uiem method over state-of-the-art approaches across different networks.

### Assessing the distinctiveness of node rankings

We analyze the distinctiveness of node ranks generated by several baseline indexing techniques, and the proposed uiem method. Monotonicity quantifies the extent to which an indexing method distinguishes each individual node within the network. Following the concept introduced by^67^, monotonicity is denoted as $M_c(I)$, where *I* refers to the sequence of node rankings generated by a specific indexing measure and is defined as:

10 $$\begin{aligned} M_c(I) =\Big [1-\frac{\sum ^N_{i\in I} N_i(N_i-1)}{N(N-1)}\Big ] ^2. \ \end{aligned}$$

Here, *N* denotes the total number of nodes in the network, while $N_i$ represents the count of nodes that share the same ranking score *i*. The monotonicity score $M_c(I)$ ranges between 0 and 1, where a value of 0 indicates complete similarity in rankings (i.e., many nodes share the same score), and a value of 1 reflects fully distinct rankings for all nodes. The computed monotonicity scores for the six indexing methods are summarized in Table 4. Compared to other indexing approaches, uiem produces a distinct and consistent ranking, as evidenced by the experimental results.

### Exploring the relationships between different indexing techniques

Here, we have assessed the performance of the proposed approach uiem with various state-of-the-art approaches such as wdc, wbc, wcc, wec, wks and the recent indexing approaches. Here, we focus on the correlation between each approach by considering the W-SIR ranking score as the ground truth. Considering these factors, we experimented and presented the results, as shown in Table 5. The experiment’s outcome shows that wbc, wcc have less rank correlation with our proposed approach. The methods wks and wce has average performance with the uiem approach. The pair (wdc, uiem) and (nsd, uiem) exhibits the highest average correlation of 0.9126 and 0.9678 respectively, outperforming all other indexing measure combinations. The results indicate a strong ranking correlation between uiem and recent indexing measure nsd, confirming the effectiveness of our proposed method when integrated with different network measures, as evidenced by this experiment.

### Examining the $\eta \%$ improvement

Here, we observe the $\eta \%$ improvement of the proposed uiem compared to the various centrality approaches. The percentage gain of the proposed method over existing baseline approaches is denoted by $\eta _\phi (\%)$ and is defined as follows:

11 $$\begin{aligned} \eta _\phi (\%) = {\left\{ \begin{array}{ll} \frac{\tau _{\text {uiem}} - \tau _\phi }{\tau _\phi } \times 100, & \text {if } \tau _\phi > 0 \\ \frac{\tau _{\text {uiem}} - \tau _\phi }{-\tau _\phi } \times 100, & \text {if } \tau _\phi < 0 \\ 0, & \text {if } \tau _\phi = 0 \end{array}\right. } \end{aligned}$$

Here, $\tau _{\text {uiem}}$ denotes the Kendall’s $\tau$ correlation coefficient between the ranking generated by the proposed *uiem* method and the ground-truth ranking obtained using the W-SIR model. $\tau _\phi$ represents the Kendall’s $\tau$ value between any baseline indexing method $\phi$ and the W-SIR model. The metric $\eta _\phi (\%)$ quantifies how much the proposed method improves upon or falls short of each baseline. A value of $\eta _\phi = 0$ implies that the proposed approach performs equivalently to the baseline method. A positive value ($\eta _\phi > 0$) indicates that the proposed method outperforms the baseline, while a negative value ($\eta _\phi < 0$) signifies a degradation in performance relative to the baseline. Figure 4 illustrates the percentage improvement $\eta _\phi (\%)$ of the proposed approach over various baseline methods across the listed complex networks. Notably, the values of $\eta _\phi (\%)$ for C(wbc) and C(wcc), when compared to their respective baselines wbc and wcc, shows low rank correlation across all networks. In the case of the ICCSS17, SFHH and Japanese Macaques networks, the *C* (*wcc*) shows low rank correlation performance. But in case of ANU-residence, Adolescent Health networks and High School *C*(*wbc*) show low correlation performance. However, among the recent indexing measures, nsd shows a comparatively stronger correlation, coming closer to the performance of uiem. Overall, the experimental results validate that the proposed *uiem* method consistently outperforms traditional benchmark techniques in terms of ranking correlation improvement, as quantified by the $\eta _\phi (\%)$ metric.

It is worth noting that Kendall’s $\tau$ and $\eta \%$ evaluate the consistency between node influence rankings and the SIR spreading results rather than the absolute outbreak size. Although varying the infection probability $\beta$ affects the scale of diffusion, the relative ordering of influential nodes tends to remain stable within the considered range of $\beta$. As a result, the correlation values appear relatively stable across different infection probabilities. Finally, we emphasize that four distinct sets of experiments were conducted, each consistently demonstrating the superior performance of the proposed *uiem* framework over a wide range of benchmark centrality and indexing techniques. An additional strength of *uiem* is its computational efficiency. Unlike many existing diffusion-based or distance-driven centrality models, *uiem* does not rely on global shortest-path computations or iterative parameter tuning, which are typically expensive in large networks. Most components of *uiem* including weighted degree, entropy, and structural reinforcement, operate in linear time with respect to the number of edges. The two-hop influence term adds a local quadratic dependency on node degrees, yet its complexity is still reasonable for sparse real-world networks. This makes *uiem* quite scalable in reality.

### Ablation study of uiem components

To analyze the marginal contribution of each component in the proposed *uiem* framework, we conducted an ablation study by systematically removing individual components and evaluating performance using the Kendall $\tau$ correlation coefficient under the W-SIR spreading model.

The final *uiem* score integrates five components: normalized $I_1$, normalized $I_2$, $lsri_{deg}$, $lsri_{wgt}$, and *dwf*. To assess the necessity of each component, we constructed multiple variants of the model by removing one component at a time and computed the corresponding Kendall $\tau$ values across all eight datasets considered in this study.

Table 6 presents the ablation results. The full *uiem* model consistently achieves the highest or near-highest Kendall $\tau$ values across all datasets, demonstrating the effectiveness of integrating multiple structural and diffusion-based components.

The impact of removing individual components varies across datasets, indicating that the contribution of each component is *network-dependent*. For instance, in the ICCSS17 dataset, removing the *dwf* component results in the largest performance drop (from 0.8463 to 0.7580), highlighting the importance of combining interaction strength and diversity in this network. In contrast, for the ANU-residence dataset, removing $I_2$ leads to a more noticeable reduction, emphasizing the role of multi-hop influence in networks with richer connectivity patterns.

An interesting observation arises in the ICCSS17 dataset, where removing $I_1$ and $I_2$ produces nearly identical Kendall $\tau$ values. This behavior suggests that, in certain network structures, local (one-hop) and second-order (two-hop) neighborhood influences may exhibit similar patterns, leading to partially overlapping contributions. Such similarity is likely due to relatively homogeneous connectivity or limited expansion in higher-order neighborhoods. However, this does not imply complete redundancy, as small differences are still observed when higher precision is considered.

Furthermore, removing $lsri_{deg}$ and $lsri_{wgt}$ leads to consistent performance degradation across most datasets, confirming their role in capturing structural reinforcement and neighborhood cohesiveness. Similarly, the removal of *dwf* significantly impacts performance in several datasets (e.g., ICCSS17, SFHH, Village), demonstrating the importance of integrating interaction diversity with connection strength.

Overall, the ablation study confirms that while certain components may exhibit overlapping effects in specific network structures, each component contributes unique and complementary information. The combined use of all five components enables the *uiem* model to effectively capture diffusion potential, structural reinforcement, and interaction diversity, resulting in improved and robust influence estimation across diverse real-world networks.

**Table 6:** Ablation study of uiem components using Kendall $\tau$ correlation under the W-SIR model.

Model variant	ICCSS17	ANU-residence	SFHH	Caenorhabditis elegans	Japanese Macaques	Adolescent health	High school	Village
Full uiem	0.8463	0.8623	0.7387	0.7042	0.8336	0.7042	0.8429	0.5935
Without $I_1$	0.8087	0.8320	0.7235	0.5740	0.8197	0.6039	0.8351	0.4991
Without $I_2$	0.8087	0.8315	0.7238	0.6544	0.8195	0.5772	0.8291	0.4986
Without $lsri_{deg}$	0.8244	0.8086	0.7293	0.4627	0.7885	0.4856	0.7858	0.4972
Without $lsri_{wgt}$	0.8109	0.8203	0.7217	0.5429	0.8281	0.5083	0.8279	0.4988
Without *dwf*	0.7580	0.8429	0.6000	0.5632	0.8207	0.5496	0.8319	0.2291

## Conclusions

The current study focuses on the key limitations in analyzing various complex networks, particularly the oversimplification introduced by unweighted network models in studying influential node identification in various real-world application scenarios. By incorporating interaction frequency as edge weights/ edge weights directly extracted from the raw dataset, we proposed the uiem approach, which effectively captures real-world contact network’s/ social networks structural and interaction dynamics. After experimenting with eight various network datasets, uiem consistently outperformed benchmark techniques currently in use in identifying the most influential nodes with significantly higher accuracy. The algorithm’s design also ensures computational efficiency by avoiding distance-based measures, making it well-suited for large-scale and real-time network analysis. These findings of our current study highlight the potential of uiem as a robust tool for applications such as epidemic modeling, information dissemination, and targeted interventions in complex systems.

Future work can explore its extension to dynamic or temporal networks and validate its applicability in diverse real-world domains. Although the present study focuses on undirected weighted networks, many real-world social and information diffusion networks exhibit directional relationships. Extending the proposed UIEM framework to directed weighted graphs by incorporating in-degree and out-degree based influence propagation and direction-aware multi-hop interactions remains an important direction for future research.

## Data Availability

The datasets analyzed during the current study are publicly available at the following link: https://networkrepository.com/ and https://www.sociopatterns.org/datasets
